# Identification of Putative Non-Substrate-Based XT-I Inhibitors by Natural Product Library Screening

**DOI:** 10.3390/biom10101467

**Published:** 2020-10-21

**Authors:** Thanh-Diep Ly, Anika Kleine, Bastian Fischer, Vanessa Schmidt, Doris Hendig, Joachim Kuhn, Cornelius Knabbe, Isabel Faust

**Affiliations:** Institut für Laboratoriums- und Transfusionsmedizin, Herz- und Diabeteszentrum NRW, Universitätsklinik der Ruhr-Universität Bochum, Georgstraße 11, 32545 Bad Oeynhausen, Germany; tly@hdz-nrw.de (T.-D.L.); ankleine@hdz-nrw.de (A.K.); bfischer@hdz-nrw.de (B.F.); vschmidt@hdz-nrw.de (V.S.); dhendig@hdz-nrw.de (D.H.); jkuhn@hdz-nrw.de (J.K.); cknabbe@hdz-nrw.de (C.K.)

**Keywords:** library screening, amphotericin B, celastrol, fibrosis, molecular docking, miRNA-21, natural products, TGF-β1, xylosyltransferase

## Abstract

Fibroproliferative diseases are characterized by excessive accumulation of extracellular matrix (ECM) components leading to organ dysfunction. This process is characterized by an increase in myofibroblast content and enzyme activity of xylosyltransferase-I (XT-I), the initial enzyme in proteoglycan (PG) biosynthesis. Therefore, the inhibition of XT-I could be a promising treatment for fibrosis. We used a natural product-inspired compound library to identify non-substrate-based inhibitors of human XT-I by UPLC-MS/MS. We combined this cell-free approach with virtual and molecular biological analyses to confirm and prioritize the inhibitory potential of the compounds identified. The characterization for compound potency in TGF-β1-driven *XYLT1* transcription regulation in primary dermal human fibroblasts (key cells in ECM remodeling) was addressed by gene expression analysis. Consequently, we identified amphotericin B and celastrol as new non-substrate-based XT-I protein inhibitors. Their XT-I inhibitory effects were mediated by an uncompetitive or a competitive inhibition mode, respectively. Both compounds reduced the cellular *XYLT1* expression level and XT-I activity. We showed that these cellular inhibitor-mediated changes involve the TGF-β and microRNA-21 signaling pathway. The results of our study provide a strong rationale for the further optimization and future usage of the XT-I inhibitors identified as promising therapeutic agents of fibroproliferative diseases.

## 1. Introduction

The human xylosyltransferase (XT) isoforms XT-I and XT-II (EC 2.4.2.26), encoded by the *XYLT1* and *XYLT2* genes, are type II transmembrane proteins localized in the Golgi [1]. Both XTs catalyze the transfer of d-xylose from uridine diphosphate (UDP)-d-xylose onto defined serine residues on similar proteoglycan (PG) core proteins and, thus initiating a series of posttranslational modifications necessary for the transport and secretion of PGs [2], which are essential components of the extracellular matrix (ECM) [3]. Both XTs consist of a short amino-terminal region facing the cytosolic side, a single transmembrane helix, a stem region necessary for Golgi retention [1], a catalytic glycosyltransferase (GT)-A domain facing the Golgi lumen [4,5] and a C-terminal domain named Xylo_C according to the Pfam database [5,6]. XT-I and XT-II are differentially expressed in tissues and cell types due to variations in the promotor region and cellular transcriptional regulations [7,8,9,10], but the reason for the existence of these two isoforms in all higher organisms remains unknown. In spite of the intracellular localization of XT-I, the enzyme is shed from the Golgi membrane by an unknown mechanism that involves a cysteine protease [11]. The XT-I secreted can be monitored by its activity in the human serum or cell culture supernatant and provides a reliable indicator of the present rate of PG biosynthesis [12,13,14]. This points to the important role of XT-I as a disease modifier in pathologies characterized by an altered PG metabolism, such as tissue fibrosis. Fibrosis is triggered by a variety of stimuli, leading to fibroblast activation and increased formation of ECM-producing myofibroblasts. Myofibroblasts, as central cellular mediators of this process, deposit excessive amounts of ECM components, such as type I collagen (*COL1A1*) or PG, that lead to stiffening and impaired tissue function [3,15,16]. Initiation of myofibroblast differentiation during fibrosis is mainly regulated by the pro-fibrotic mediator transforming growth factor-β1 (TGF-β1). Alpha smooth muscle actin (*ACTA2*), *COL1A1* and *XYLT1* expression were demonstrated to be upregulated in activated fibroblasts [17,18], while *XYLT2* mRNA expression is unaffected by TGF-β1 [17,19,20,21]. The TGF-β1-induced increase in *XYLT1* expression results in higher cellular XT-I activity and PG accumulation [3,17,18]. Therefore, elevated *XYLT1* expression is also associated with persisting fibrosis and displays a reliable marker for myofibroblast differentiation [12,17].

In addition, TGF-β1 can modulate a number of other cytokines, cellular downstream signaling pathways and microRNA (miRNA) expressions [22,23]. The miRNAs are small noncoding endogenous RNAs which bind to the 3′-untranslated regions of target mRNAs, preventing the translation or leading to the degradation of the respective mRNAs. Aberrant miRNA expression has been reported in a wide range of diseases including fibrosis [24,25]. One pathomechanism of this process involves the inhibitory SMAD7, a direct target of miRNA-21-5p (miRNA-21). Consequently, upregulation of miRNA-21 inhibits SMAD7 and results in enhanced TGF-β1 signaling in primary pulmonary and cardiac fibroblasts [26,27].

However, the broad impact of TGF-β signaling on numerous cellular functions in tissue homeostasis and disease makes developing an effective but safe TGF-β-dependent therapy challenging. Potential strategies for targeting TGF-β and its signaling pathways include focusing on modifiers and downstream products, pathway inhibition at the ligand or receptor stage and the blocking of downstream signaling components or miRNAs [28]. Therefore, targeted blocking of XT expression and activity could provide a therapeutic strategy for PG-associated diseases. To date, only substrate analogue XT inhibitors such as nucleotides and glycosaminoglycans (GAGs) have been identified, but their inhibitory activity is restricted to cell-free approaches [29,30]. The aim of this study was, therefore, to identify new non-substrate-based XT-I inhibitors from natural compounds that could potentially be suitable for therapeutic applications. 

Natural products (NPs) are the earliest forms of human medicine with strong, unique, anti-inflammatory, anticancer or neuroprotective properties [31,32]. The NPs are often more efficient than artificially made compounds if a specific biological activity, such as an enzyme inhibitor, is needed. The NPs are classified based on their origins, biological functions and structures. Plants, bacteria and fungi are a vast source of many natural drugs. With the exception of peptides and carbohydrates, the most important of these NPs are terpenoids, alkaloids, steroids, phenolic compounds, vitamins, carbocyclics and heterocyclic aromatic compounds [33].

## 2. Materials and Methods

### 2.1. Materials

The 96-well plate format compound library with NPs, pre-dissolved in dimethyl sulfoxide (DMSO, 10 mM), was obtained from Selleck Chemicals Llc (Houston, Texas, United States). Celastrol and amphotericin B were purchased from Biomol GmbH (Hamburg, Germany) and Sigma-Aldrich (St. Louis, Missouri, United States), respectively, and dissolved in DMSO to gain a 10 mM stock solution. Recombinant human TGF-β1 was purchased from Miltenyi Biotec (Bergisch Gladbach, Germany). The miRNAs, transfection reagents and media were obtained from Thermo Fisher Scientific (Waltham, Massachusetts, United States).

### 2.2. Expression of Recombinant Human XT-I in pgsA-745 Chinese Hamster Ovary Cells

The XT-deficient pgsA-745 Chinese hamster ovary (CHO) cells [34] in which XT-I expression was complemented with a full-length *XYLT1* containing plasmid (pgsA-6HisXT1-K4) [1] were cultured as described previously [11,35]. In brief, pgsA-6HisXT1-K4 cells were grown as monolayer culture in 175 cm^2^ T-flasks with 25 mL Ham’s F12 medium supplemented with 10% (*v*/*v*) fetal calf serum (FCS, Biowest, Nuaillé, France) and 400 g/L Geneticin G418 (Invivogen, Toulouse, France) under standard conditions. Upon confluence, adherent cells of two T-flasks were transferred to a 100 × 20 mm cell culture dish with 10 mL of serum-free Ham’s F12 medium. The cell culture supernatant was collected after four days and centrifuged at 250× *g* for 5 min to obtain the secreted recombinant XT-I protein.

### 2.3. Bicinchonic Acid Assay

The bicinchonic acid (BCA) assay was used to quantify the amount of protein in a sample [36]. Bovine serum albumin (Sigma Aldrich, St. Louis, Missouri, United States) was used to generate a standard curve ranging from 0 to 1000 g/L. The assay was conducted according to Smith et al. in a 96-microplate format [36]. A volume of 25 μL standard or protein sample was combined with 200 μL BCA working reagent, which was prepared by mixing 50 parts of BCA solution with 1 part of Cu^2+^ solution. The absorbance at 562 nm was measured after an incubation period of 30 min at 37 °C. The protein concentrations obtained were used for XT-I activity normalization.

### 2.4. Determination of XT-I Activity by Mass Spectrometry

The measurement of relative XT-I activity among different samples is based on the XT-catalyzed incorporation of xylose from UDP-d-xylose on a modified bikunin-derived peptide after a fixed reaction time. The assay was originally developed for the determination of XT activity in human- or cell-culture-derived samples [13] by ultra-performance liquid chromatography/electrospray ionization tandem mass spectrometry (UPLC/ESI-MS/MS). The UPLC/ESI-MS/MS XT assay was recently modified toward a XT-I selective activity determination in human serum and cell culture samples (unpublished data). The relative intensities of the xylosylated peptide species abundance obtained with the respective m/z ratio were directly proportional to the relative XT-I activity of the sample. The XT-I activity determined is expressed in arbitrary units and normalized to the total protein content per 1 mL sample volume in cell culture approaches. A calibrator probe with a fixed amount of recombinant XT-I enzyme was included in every UPLC-MS/MS XT-I assay for assay comparability across multiple runs. The assay reaction mixture (50 μL) contained 50% (*v*/*v*) of the respective XT-I enzyme solution and 50% (*v*/*v*) reaction buffer. The final XT-I assay contained 25 mM MES buffer (pH 6.5), 25 mM KF buffer, 5 mM MnCl_2_, 5 mM MgCl_2_, 7.0 μM modified acceptor peptide and 30 μM UDP-d-xylose. The sample preparation for UPLC/ESI-MS/MS was performed according to [13]. In brief, after sample incubation at 37 °C for 24 h, the reaction was stopped by heating at 99 °C for 10 min. After centrifugation (10,000× g, 10 min) the supernatant was diluted fivefold with UPLC water for analysis. All cell-based XT-I assays were carried out in three biological and three technical replicates per donor-derived primary cell culture, while the XT-I activity determination for screening or kinetic purposes was carried out at least in technical duplicates.

### 2.5. Inhibitor Screening Assay and Determination of IC_50_ Values

Inhibitor candidates from the NP compound library were screened using the UPLC/ESI-MS/MS XT-I assay with recombinant XT-I protein solution from pgsA-6HisXT1-K4 cells. In order to identify putative XT-I inhibitor compounds, equally concentrated XT-I protein solutions were preincubated with 1% (*v*/*v*) DMSO or 100 μM of each inhibitor for 1 h at room temperature (RT). Thereafter, 50% (*v*/*v*) of the XT-I enzyme mixture was supplemented with 50% (*v*/*v*) reaction buffer. The final screening assay contained 50 μM inhibitor or 0.75% (*v*/*v*) DMSO, 25 mM MES buffer (pH 6.5), 25 mM KF buffer, 5 mM MnCl_2_, 5 mM MgCl_2_, 10.5 μM modified acceptor peptide and 15 μM UDP-d-xylose. The sample preparation for UPLC/ESI-MS/MS was performed according to [13] and diluted fivefold with UPLC water for analysis. In order to determine the IC_50_ values of the inhibitor compounds identified, equally concentrated XT-I protein solutions were preincubated with DMSO or inhibitors in the concentration range of 0.02 to 150 μM for 1 h at RT. The XT-I activity of the protein-inhibitor mixtures were determined by UPLC/ESI-MS/MS as described above. The DMSO content in this enzyme-catalyzed reaction was kept constant at a final concentration of 0.75% (*v*/*v*), which corresponds to a DMSO content of 0.15% (*v*/*v*) after the fivefold dilution step with UPLC water.

### 2.6. Enzyme and Inhibition Kinetics

The mode of inhibition (MOI) study was performed by evaluating the inhibitors’ impact on the enzyme parameters’ Michaelis constant *K*_m_ and the maximum activity *V*_max_. In order to determine the enzyme and inhibition kinetics, XT-I activity determination of the recombinant XT-I protein solution with and without inhibitor supplementation was carried out in the presence of different acceptor peptide concentrations ranging from 1 to 30 μM. In addition to the modified acceptor peptide, the final reaction mixture (50 μL) contained 50% (*v*/*v*) XT-I enzyme solution with or without inhibitor at IC_50_ concentration, 25 mM MES buffer (pH 6.5), 25 mM KF buffer, 5 mM MnCl_2_, 5 mM MgCl_2_ and 15 μM UDP-d-xylose. The *K*_m_ and *V*_max_ were determined with the Michaelis–Menten equation and nonlinear regression analysis, while the slope and Y-intersection values were determined by a Lineweaver–Burk plot and simple linear regression using GraphPad Prism 8.0 (GraphPad Software, La Jolla, United States) software. The *V*_max_ values after a fixed reaction time are displayed in arbitrary units equaling the product generation per time d*P*/d*t*, while the *K*_m_ values are expressed in the unit of concentration. 

### 2.7. Displaying Putative Inhibitor Binding Sites by Molecular Docking 

The estimation and visualization of putative compound binding sites in the XT-I protein were conducted by molecular docking software AutoDock Vina [37] within UCSF Chimera 1.14 [38], a molecular visualization software. The PDB file for the three-dimensional structure of human XT-I apoprotein (PDB ID: 6FOA) as a target macromolecule template with a resolution of 1.867 Å was downloaded from the RCSB protein data bank [39] and processed with the Chimera tool Dockprep. The in silico receptor preparation, applied to extract only the protein chain of interest, was completed by removing all heteroatoms and water molecules, fixing nonstandard residues and adding hydrogen. The protonation state of the protein was adjusted to a neutral pH and the maximum energy difference was set to 3 kcal/mol. The structures of the active compounds were obtained from the PubChem database [40] using canonical SMILES then converted to a usable ligand molecule for subsequent in silico docking runs. After receptor and ligand preparation, the complete protein surface was defined as a binding site for docking analysis. All molecular docking simulations run with AutoDock Vina were executed with the identical setup described above and visualized with the ViewDock implementation. The binding positions of the UDP-d-xylose and acceptor peptide were adopted from the 2.0 Å resolved human XT-I structure (PDB ID: 6EJ7) for direct comparison of the binding position of inhibitors and substrate molecules. The amino acid sequence of the bikunin-derived acceptor peptide was transmuted to the amino acid sequence of the acceptor peptide applied for better illustration using the UCSF Chimera 1.14 application Rotamers. The chimera models (PDB ID: 6FOA) with the docked ligand positions identified by AutoDock Vina were then aligned with the acceptor peptide-modified human XT-I structure complexed with UDP-d-xylose (PDB ID: 6EJ7) utilizing the integrated application MatchMaker [41].

### 2.8. Primary Cell Culture, Treatment and Sample Preparation

Normal human dermal fibroblasts (NHDFs) from a 37- and a 50-year-old woman and a 57-year-old man were obtained commercially from Cambrex (Walkerville, United States) and Coriell (New Jersey, United States). The NHDFs were cultured in phenol red-free Dulbecco’s modified Eagle’s medium (DMEM; Thermo Fisher Scientific, Waltham, Massachusetts, United States) supplemented with 10% (*v*/*v*) FCS, 4 mM l-glutamine and 100,000 *U*/L penicillin, 100 mg/L Streptomycin, 0.25 mg/L amphotericin B (PAN Biotech, Aidenbach, Germany) at 37 °C, 5% CO_2_. The medium was changed twice a week until the cells reached 80% confluency. The NHDFs were subcultured with an expansion ratio of 1:3 using 0.05% trypsin (PAN Biotech, Aidenbach, Germany) and used until the ninth passage.

A former established cell culture model was utilized to investigate the effect of putative XT-I inhibitors on NHDFs [17]. The inhibition studies were carried out with final inhibitor concentrations of 0.5, 1.0, 2.0 or 4.0 μM diluted in fully supplemented DMEM. In brief, unless otherwise stated, NHDFs were cultivated with a low density of 50 cells per mm^2^ on a hard tissue culture substrate (100 × 20 mm dish) in fully supplemented DMEM with or without additional TGF-β1 (5 μg/L) for 24 h, promoting their transdifferentiation into proto-myofibroblasts. Thereafter, cells were treated with celastrol or amphotericin B in the presence or absence of TGF-β1 (5 μg/L) for an additional 48 h. Negative controls treated with solvent only were included for every sampling time. The final DMSO content of 0.04% (*v*/*v*) was kept constant in all inhibition experiments.

The NHDFs (2.9×10^5^ per 60 mm dish) were maintained in antibiotic-free medium and reverse transfected with miRNA-21 mimic or negative control miRNA using Lipofectamine 2000 transfection reagent to analyze miRNA-mediated effects. After 24 h, the transfection mixture containing a final miRNA concentration of 100 nM was replaced with serum-free DMEM supplemented with TGF-β1 (10 μg/L) for 48 h until cell lysis.

The cell culture supernatant was collected to analyze the extracellular XT-I activity, whereas the same cell culture monolayer was lysed using a fixed amount of 0.75 mL Nonidet P-40 (NP-40)-buffer (50 mM TRIS, 150 mM NaCl, 1% NP-40, pH 7.8) for the analysis of intracellular XT-I activities. After sample centrifugation (10,000× g, 10 min, 4 °C), the supernatant contained the intracellular XT-I protein. The lysates were also used for the BCA assay. The cellular XT-I activities were measured 48 h after inhibitor treatment (48 h) or an additional 48 h after inhibitor removal (96 h). 

All cell culture experiments were conducted in biological duplicate or triplicates per number n of donor-derived primary cultures as indicated.

### 2.9. Cell Proliferation Assay

The tetrazolium salt WST-1 (Roche, Basel, Swiss) was used for the spectrophotometric quantification of cell proliferation in response to various inhibitor concentrations. The cell proliferation assay was carried out according to the manufacturer’s instructions in a 96-well tissue culture plate with 1700 cells per cavity. Based on the inhibition studies, the cells were cultured in fully supplemented DMEM for 24 h then treated with celastrol or amphotericin B for an additional 48 h. The WST-1 reagent was added to the cell culture supernatant 4 h before the end of the treatment. The absorptions at 440 and 590 nm were measured at timepoints 0, 1, 2, 3 and 4 h after the addition of WST-1.

### 2.10. Nucleic Acid Extraction and Synthesis of Complementary DNA

The RNA extraction from cell lysates and cDNA synthesis for mRNA and miRNA expression analysis were performed as described previously [7,42].

### 2.11. mRNA and miRNA Expression Analyses

Quantitative real-time polymerase chain reaction (qRT-PCR) analysis using a LightCycler 480 Instrument II system (Roche, Basel, Swiss) was performed as previously described [7]. The qRT-PCR experiments were conducted with three biological and three technical replicates per donor-derived primary cell culture, unless otherwise stated. The intron-spanning primer sequences used are listed in [7] and Table 1. The geometric mean of the expression levels of the housekeeping genes *SDHA*, *RPL13A* and *B2M* were calculated for expression normalization. The relative target gene mRNA expressions were determined by the ΔΔC_T_ method considering the PCR efficiency [43]. All normalized gene expression levels shown were referred to the respective target gene expression of one primary cell culture sample for the relative comparison of multiple biological samples per experiment and figures.

The TaqMan advanced miRNA Assay (Thermo Fisher Scientific; Waltham, Massachusetts, United States) was performed, according to the manufacturer’s instructions, for the miRNA expression analyses. The primer assays miRNA-21 (477975_mir) and miRNA-191 (477952_mir) were used. The expression level of miRNA-21 determined was normalized to internal housekeeping gene expression of miRNA-191 via the ΔΔC_T_ method.

### 2.12. Statistical Analysis

All data are presented as mean values ± standard error of the mean (SEM). The assumption of normality was checked visually via frequency distribution histogram and by computing a Shapiro–Wilk normality test. Due to the lack of Gaussian distribution, the statistical analysis between experimental conditions was evaluated by a nonparametric two-tailed Mann–Whitney U test using GraphPad Prism 8.0 (GraphPad Software, La Jolla, CA, USA) software. Probability P values of less than 0.05 were considered statistically significant. P values are indicated with asterisks and horizontal lines connecting the bars being compared. Asterisks shown directly above the error bars of the treatment group indicate statistical differences between treated and untreated groups.

## 3. Results

### 3.1. Identification of Putative Non-Substrate-Derived XT-I Inhibitors Celastrol and Amphotericin B

An NP library of 96 compounds was screened to identify a novel non-substrate-based XT-I inhibitor. The inhibitor screening and enzymatic activity determination were performed using pgsA-6HisXT1-K4 cell-derived XT-I protein as an enzyme source and an adapted in vitro UPLC/ESI-MS/MS XT assay for selective XT-I activity determination. The compounds (Supplementary Table S1) were applied at 50 μM, whereby the impact of 0.05 to 0.15% (*v*/*v*) DMSO on the ionization process in ESI/MS [44] was initially excluded experimentally (unpublished data). Four compounds in this preliminary screening were found to exhibit an XT-I activity reduction to less than 50% of the negative control, which was set to 100% (Table 2).

**Figure 1:**
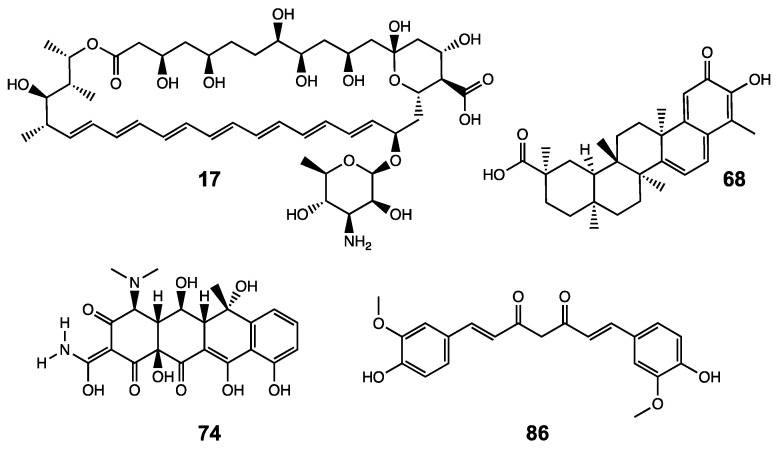
Structures of the compounds identified from the initial library screening assay. Amphotericin B (17), celastrol (68), oxytetracycline (74) and curcumin (86) are shown. Structures were drawn using the software ChemDraw 19.1 and retrieved from the chemical structure database ChemSpider [45].

The compounds identified were amphotericin B (17), celastrol (68), oxytetracycline (74) and curcumin (86). The chemical structures of these hit compounds are shown in Figure 1.

We considered the literature-based screening results and structural features of the compounds identified to decide whether they were suitable for further cell-based analysis. Detailed information is described in the discussion part. Additionally, we performed an in silico blind docking analysis to correlate the experimental inhibition results for compounds 17, 68, 74 and 86 with the predicted ones by USCF Chimera 1.14 [38] AutoDock Vina [37]. We used the respective inhibitor structures as the ligand and apoprotein structure of XT-I previously determined by Briggs et al. [5] as the receptor input to perform a docking screen. The docking program used generates a number of potential ligand conformations and orientations relative to the XT-I protein. Those ligand-protein models (chimera model) predicted are ranked by a scoring function according to the likelihood of their binding interaction. A low negative score indicates a stable system and, thus, a likely binding interaction [37]. A summary of the docking results of the virtual screen is shown in Table 3. 

The virtual analysis of the two most favored inhibitor docking modes by USCF Chimera 1.14 [38] AutoDock Vina [37], ranked by the values of their scoring function, showed the most negative values for the two compounds amphotericin B (17) and celastrol (68). Compound 22, which did not possess any inhibitory effect on XT-I activity in the primary assay (Supplementary Table S1), showed the lowest AutoDock Vina docking score (Table 3). Since the value expressed by the docking score is a sum of contributions from different energy terms, such as electrostatic, H-bond, Van-der-Waals or conformation energy, we noticed the strongest discrepancy with compound 74 by comparing the ranked docking scores (Table 3) with the ranked experimental results of the initial compound screening (Table 2). Taking the results of the experimental screen, the virtual screen and the possibility of false-positive results [46], the compounds 74 and 86 were not considered for further analysis.

**Figure 2:**
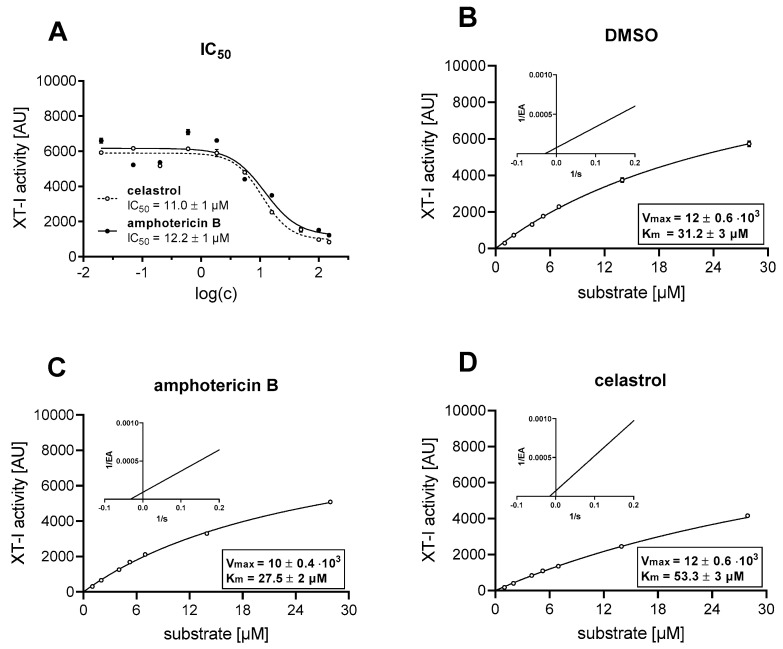
Enzyme and inhibition kinetics. The enzyme activity (EA) illustrated corresponds to the generated xylosylated-acceptor peptide measured by the UPLC-ESI-MS/MS XT-I assay after a fixed time point and, therefore, is expressed in arbitrary units (AU) detected at a certain retention time. (**A**) Determination of IC_50_ values of compounds 17 (amphotericin B) and 68 (celastrol) at 15 μM UDP-d-xylose, 10.5 μM acceptor peptide and various inhibitor concentrations (c). The values shown are mean ± SEM of duplicate data points per experiment. (**B**–**D**) Michaelis–Menten and Lineweaver–Burk plots for the modified acceptor peptide (substrate, s) at 15 μM UDP-d-xylose. Values are means ± SEM of triplicate data points per experiment.

The two remaining compounds 17 and 68 were subjected to an IC_50_ value determination assay under the same assay conditions as the primary screening assay, except for the use of different inhibitor concentrations in the pre-incubation step with XT-I enzyme, to verify the experimental results of the preliminary screening. Consistent with the XT activity reduction observed previously (Table 2), the two compounds 17 and 68 showed comparable IC_50_ values of 12.2 ± 1 μM (amphotericin B) and 11.0 ± 1 μM (celastrol) (Figure 2A).

We next performed kinetic studies to investigate the MOI of these potent compounds and correlated the data with the chimera models obtained with Autodock Vina. The enzyme activity of CHO cell-derived XT-I protein samples were measured after incubation with the inhibitor compounds or DMSO for 1 h. The reaction was started by incubation of 50% (*v*/*v*) of the CHO-inhibitor mixture with 50% (*v*/*v*) reaction buffer containing UDP-d-xylose and various acceptor peptide concentrations, each at 37 °C. The Michaelis–Menten and Lineweaver–Burk plots for the modified acceptor peptide are shown in Figure 2. The Michaelis–Menten constant *K*_m_, maximum enzyme activity *V*_max_ or Y-intersect and slope values derived from this analysis are summarized in Supplementary Table S2.

The compound amphotericin B (17) used at IC_50_ concentration resulted in a decreased K_m_ value and a concomitant decrease in the V_max_ value compared to the assay containing DMSO, indicative of an uncompetitive MOI regarding the acceptor peptide. This mechanism of inhibition is characterized by changing the Y-intercept but not the slope value in the Lineweaver–Burk plot (Figure 2B,C; Supplementary Table S2).

The usage of compound celastrol (68) at the IC_50_ concentration resulted in an increased K_m_ value compared to the approach containing DMSO. The V_max_ value determined did not differ between the celastrol and assay containing DMSO. Regarding the parameters extracted from the Lineweaver–Burk plot, the value of the Y-intercept was higher in the assay containing celastrol, while the slope value did not vary considerably from the assay containing DMSO (Figure 2B,D; Supplementary Table S2). The results of the Michaelis–Menten and Lineweaver–Burk plots are indicative of a competitive mechanism of inhibition. 

**Figure 3:**
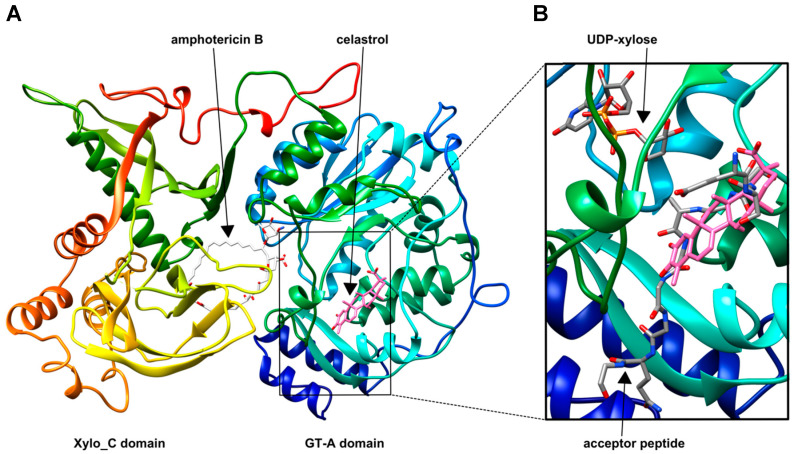
Three-dimensional structure of human XT-I complexed with the compounds amphotericin B (**17**) and celastrol (68). (**A**) Crystal structure of human XT-I [5], rainbow colored from the N terminus (blue) to C terminus (red), complexed with the chimera models #2 of compounds 17 (amphotericin B, white colored) and 68 (celastrol, pink colored). (**B**) UDP-d-xylose, modified acceptor peptide (atoms: C (grey), N (blue), O (red), P (orange)) and docked orientation of compound 68 (celastrol, pink) are shown in the stick representation.

In comparison with the most favored docking modes offered by USCF Chimera 1.14 [38] AutoDock Vina [37] (Table 3), the chimera models amphotericin B #2 and celastrol #2 were able to confirm the experimentally approved situation exactly (Figure 3).

It could be observed by analyzing the binding zone (< 5.0 Å) of amphotericin B #2 in the XT-I protein complexed with UDP-d-xylose and modified acceptor peptide that the binding of the acceptor peptide was unaffected by amphotericin B binding. Furthermore, an overlap of the amphotericin B binding site and UDP-d-xylose could be detected (Supplementary Figure S1). As illustrated in Figure 3, celastrol might bind to the active site of the XT-I, preventing the acceptor peptide from binding. Binding zone (< 5.0 Å) analysis of celastrol #2 reflected a spatial proximity to the UDP-d-xylose molecule, as was shown for the modified acceptor peptide (unpublished data). The consistency of both experimental and in silico results indicate that amphotericin B is an uncompetitive inhibitor toward the acceptor peptide substrate, while celastrol seems to exhibit a competitive binding mode in this context.

### 3.2. Celastrol and Amphotericin B Inhibit Cellular Proliferation Dose-Dependently

**Figure 4:**
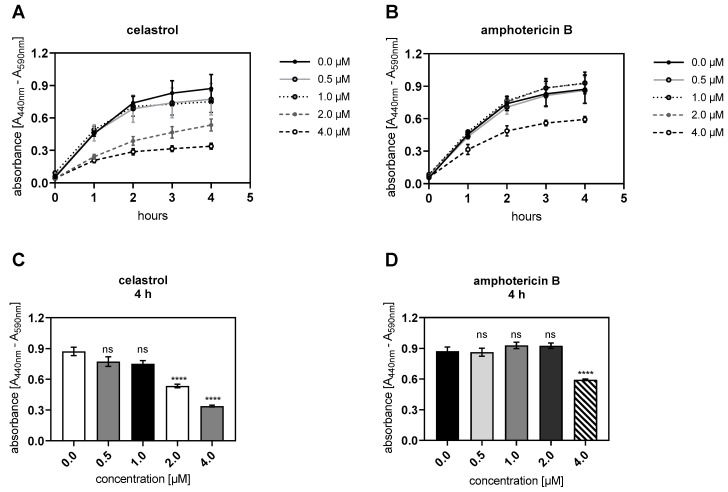
Celastrol and amphotericin B treatment inhibited the NHDF proliferation in a dose-dependent manner. Human primary dermal fibroblasts (n = 2) were cultured the day before the experiment for measuring the cell proliferation in response to different inhibitor treatments. Cells were treated with vehicle (0.0 μM) or 0.5, 1.0, 2.0 and 4.0 μM (**A**) celastrol or (**B**) amphotericin B for 48 h. Cellular proliferation was detected by the addition of tetrazolium salt WST-1 to the cell culture supernatants at the 44 h timepoint and measured 0, 1, 2, 3 and 4 h post-supplementation. The absorbance measured correlates directly to the number of cells viable after the inhibitor treatment. (**C**,**D**) Bar chart display of the WST-1 assay results measured 4 h post supplementation of NHDFs with WST-1. Data are means ± SEM of five biological and one technical replicate per experiment. Mann-Whitney U test: not significant (ns), *p* < 0.0001 (****).

The cytotoxic effects of amphotericin B and celastrol on the proliferation of NHDFs was tested with the WST-1 assay. Both compounds in these experiments were used at concentrations ranging from 0.0 to 4.0 μM (Figure 4).

We found significantly decreased cell proliferation in cells treated with 2.0 and 4.0 μM celastrol (both *p* < 0.0001) compared to the control after 48 h of inhibitor incubation (Figure 4A,C). The usage of 4.0 μM amphotericin B also reduced the proliferation of NHDFs significantly (*p* < 0.0001) compared to the control (Figure 4D).

Taking these results together, the treatment of NHDFs with 0.5 and 1.0 μM celastrol or the usage of amphotericin B at a concentration range of 0.5 to 2.0 μM for 48 h did not influence cellular proliferation. Thus, these inhibitor concentrations and incubation durations could be used for the next cell-based experiments.

### 3.3. Dual Effect of Putative XT-I Inhibitors on XYLT1 mRNA Expression and XT Activity of NHDF

**Figure 5:**
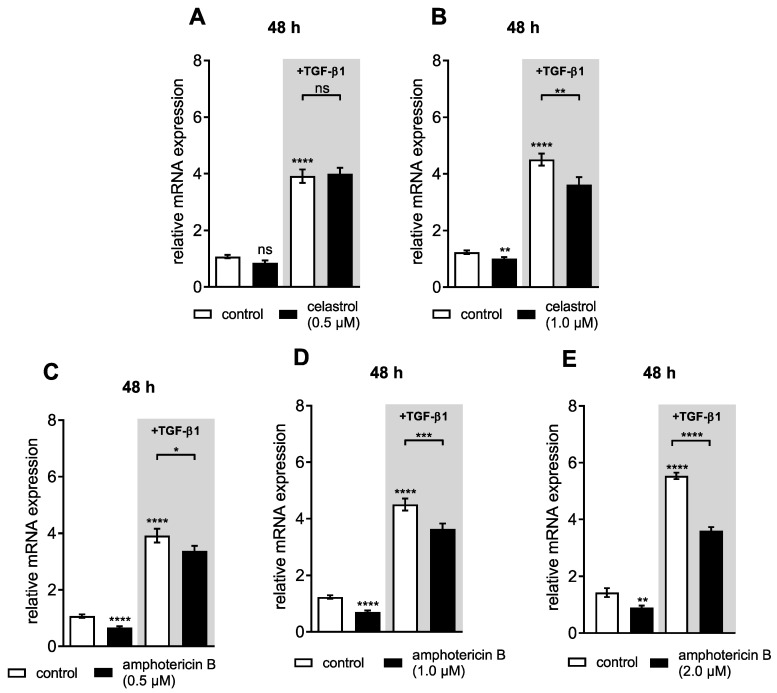
Celastrol and amphotericin B treatment reduced the basal and TGF-β1-mediated *XYLT1* mRNA expression in NHDFs. Human primary dermal fibroblasts (*n* = 3) were cultured the day before the experiment. Cells were treated with vehicle (control), (**A**,**B**) celastrol (0.5 or 1.0 μM) or (**C**–**E**) amphotericin B (0.5, 1.0 or 2.0 μM) for 48 h with or without additional TGF-β1 (5 μg/L) supplementation. Relative *XYLT1* mRNA expression levels were analyzed by qRT-PCR. Data are means ± SEM of three biological and three technical replicates per experiment. Mann–Whitney U test: not significant (ns), *p* < 0.05 (*), *p* < 0.01 (**), *p* < 0.001 (***), *p* < 0.0001 (****).

Previous studies have shown that the cellular XT-I activity is regulated on the transcriptional level [7]. After demonstrating that the DMSO content of 0.04% (*v*/*v*), the celastrol concentrations of 0.5 and 1.0 μM and the amphotericin B usage of 0.5 to 2.0 μM are well tolerated by NHDFs, we wanted to evaluate the effects of the putative XT-I inhibitors on the *XYLT1* mRNA expression and cellular XT-I activity. Consequently, NHDF cells were cultured as a monolayer in a previously established fibrosis cell culture model [17] and treated subsequently with celastrol or amphotericin B in the presence of fibrotic mediator TGF-β1, which had been previously shown to increase the *XYLT1* mRNA expression in fibroblasts [17,18]. The *XYLT1* mRNA expression levels were quantified 48 h posttreatment by qRT-PCR analysis (Figure 5). 

The NHDFs showed an increase in *XYLT1* mRNA expression after 48 h of TGF-β1 treatment (3.7 ± 0.3-fold, *p* < 0.0001; Figure 5A–D). The usage of 0.5 μM celastrol neither changed the basal *XYLT1* mRNA expression nor effected the TGF-β1-mediated *XYLT1* mRNA expression increase in NHDFs (Figure 5A). However, a significant reduction in both basal and TGF-β1-mediated *XYLT1* mRNA expression was observed with 1.0 μM celastrol (0.8 ± 0.06-fold, *p* = 0.005 and 0.8 ± 0.07-fold, *p* = 0.01) (Figure 5B). Regarding the mRNA expression of isoform *XYLT2* in NHDFs, which was unaffected by TGF-β1 supplementation, the addition of 1.0 μM celastrol in the presence of TGF-β1 did not alter the *XYLT2* mRNA expression level in these cells (Supplementary Figure S2A).

The amphotericin B treatment of NHDFs at concentrations of 0.5 and 1.0 μM significantly decreased the basal *XYLT1* mRNA expression (0.6 ± 0.06-fold and 0.6 ± 0.05-fold, respectively, both *p* < 0.0001). Furthermore, we observed a significant reduction of the TGF-β1-induced *XYLT1* mRNA expression in NHDFs due to the supplementation of amphotericin B at concentrations of 0.5 and 1.0 μM (0.9 ± 0.07-fold, *p* = 0.04 and 0.8 ± 0.06-fold, *p* = 0.0003, respectively) (Figure 5C,D). The *XYLT1* mRNA expression in cells 48 h after supplementation with amphotericin B at a concentration of 2.0 μM was considerably reduced in comparison to the mRNA expression of controls treated with DMSO (0.6 ± 0.09-fold, *p* = 0.005). The TGF-β1 treatment of NHDFs increased the basal *XYLT1* mRNA expression level significantly (4.6 ± 0.2-fold, *p* < 0.0001; Figure 5E). This TGF-β1-mediated effect on the *XYLT1* mRNA expression level was remarkedly reduced by 2.0 μM amphotericin B (0.7 ± 0.03-fold, *p* < 0.0001) (Figure 5E). In concordance with previous results [7], the isoform *XYLT2* mRNA expression level was unaffected by the TGF-β1 treatment. Furthermore, the basal *XYLT2* mRNA expression of cells treated with TGF-β1 did not differ from that of those additionally treated with amphotericin B (Supplementary Figure S2B).

Together, these results indicate that amphotericin B and celastrol were not only capable of interfering with the XT-I protein itself but could also regulate cellular *XYLT1* mRNA expression due to a yet unknown mechanism.

### 3.4. Inhibitor-Induced mRNA Expression Changes Lead to Decreased XT-I Protein Expression in NHDF

In order to examine whether the inhibitor-mediated reduction of *XYLT1* gene expression correlates with changes in the extracellular and intracellular XT-I activity of NHDFs, we usually determine the cellular XT-I activity by UPLC-MS/MS under the same experimental conditions as the gene expression analysis. This time, however, we had to consider that the exogenously supplemented inhibitor compounds could change the extracellular XT-I activity measured (Supplementary Figure S3). In comparison to the extracellular XT activity of untreated cells, the supplementation of celastrol for 48 h at a concentration of 0.5 μM led to a significant reduction of the XT-I activity (0.8 ± 0.07-fold, *p* = 0.03) (Supplementary Figure S3). Under the same experimental conditions, no *XYLT1* mRNA expression changes were present in cells treated with celastrol (Figure 5A). These results revealed that the XT-I activity measured in the presence of the exogenous supplemented inhibitor was artificial and did not reflect the transcriptional state of the cell.

**Figure 6:**
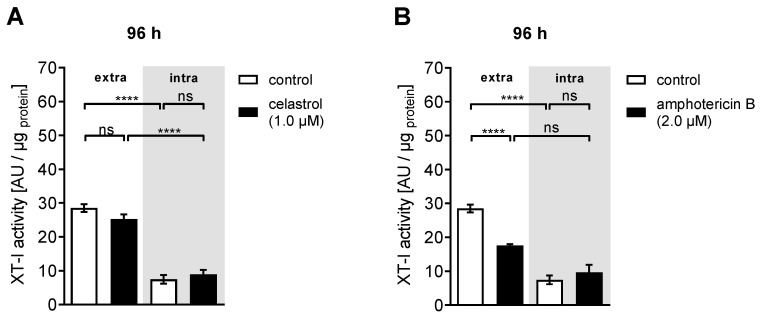
Amphotericin B was capable of reducing the extracellular XT-I activity in a fibrosis cell culture model. Human primary dermal fibroblasts (*n* = 3) were cultured in media supplemented with TGFβ1 (5 μg/L) for 24 h in order to enhance their myofibroblast differentiation. Thereafter, cells were treated with DMSO (control, white), (**A**) 1.0 μM celastrol or (**B**) 2.0 μM amphotericin B containing media supplemented with TGF-β1 (5 μg/L) for 48 h. Cells were washed twice with 1x PBS and cultured in TGF-β1- and inhibitor-free media for an additional 48 h (96 h). The intracellular XT-I activity (intra, grey) was determined from the cell lysates and the corresponding supernatants were utilized for extracellular XT-I activity (extra) determination by UPLC-ESI-MS/MS XT-I assay. The XT-I activity is expressed in arbitrary units (AU) per μg of protein in a 1 mL sample. Data are means ± SEM of three biological and three technical replicates per experiment. Mann–Whitney U test: not significant (ns), *p* < 0.0001 (****).

In order to address this limitation, we decided to change the experimental setup, allowing us to determine the cellular XT-I activity changes that were caused by former inhibitor-mediated changes on the *XYLT1* mRNA expression in NHDFs by UPLC/ESI-MS/MS. The results of this XT-I activity determination after simultaneous treatment of cells for 48 h with TGF-β1 and the highest tolerated inhibitor concentrations, followed by an incubation of the NHDFs in inhibitor-free media for an additional 48 h, are shown in Figure 6. 

A slight but not significant decrease in extracellular XT activity was observed in the NHDFs upon treatment with celastrol (0.9 ± 0.06-fold, *p* = 0.06) (Figure 6A). By contrast, the usage of amphotericin B resulted in a statistically significant reduction of extracellular XT-I activity (0.6 ± 0.03-fold, *p* < 0.0001) (Figure 6B). The celastrol and amphotericin B treatments did not result in any detectable changes in the intracellular XT activities of NHDFs compared to control cells treated with DMSO. It can therefore be concluded that the celastrol- and amphotericin B-mediated changes to the *XYLT1* mRNA expression observed previously correlate with the extracellular XT-I activity changes, especially for 2 μM amphotericin B, while the intracellular XT activity seemed to return to a homeostatic state during the cell incubation in inhibitor-free media for 48 h.

### 3.5. Celastrol-Induced XYLT1 Suppression Might Be Mediated by the miRNA-21 Pathway 

**Figure 7:**
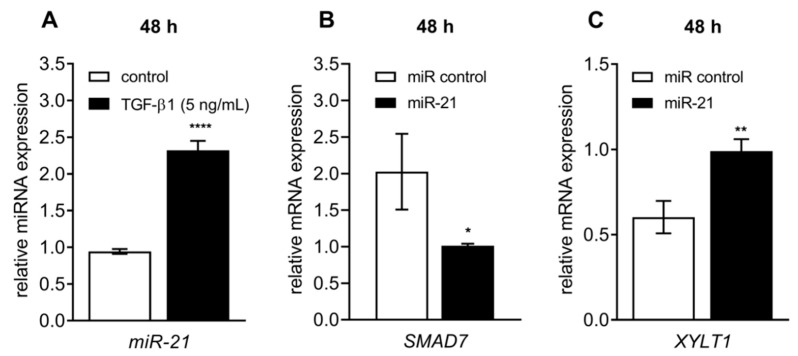
Reciprocal regulation of the *XYLT1* and *SMAD7* mRNA expression by miRNA-21. (**A**) Human primary dermal fibroblasts (*n* = 3) were cultured the day before the experiment. Cells were serum-starved for 24 h and treated with or without TGF-β1 (5 µg/L) for 48 h. Relative miRNA-21 (miR-21) expression levels were analyzed by qRT-PCR using Taqman probes. (**B**,**C**) The NHDFs (*n* = 3) were transfected with an miRNA-21 mimic (miR-21) or negative control miRNA mimic (miR control). After 24 h, cells were incubated in media supplemented with TGF-β1 for 48 h. Relative *XYLT1* and *SMAD7* mRNA expressions were analyzed by qRT-PCR. Data shown are means ± SEM for two biological and three technical replicates per experiment. Mann–Whitney U test: *p* < 0.05 (*), *p* < 0.01 (**), *p* < 0.0001 (****).

After showing that celastrol exerts a suppressive effect on *XYLT1* mRNA expression, we wanted to evaluate a putative cellular pathway underlying this regulation. miRNA-21 plays a crucial role in TGF-β1/Smad pathway-mediated tissue fibrosis [24,47]. Earlier studies by Cheng et al. showed the involvement of the miRNA-21/ERK pathway in celastrol-mediated anti-fibrotic effects in murine cardiac fibroblasts [48]. In concordance with the study by Ni et al. [49], Cheng et al. demonstrated a celastrol-induced suppression of miRNA-21 expression [48]. Based on these findings, we wanted to verify the impact of the miRNA-21 pathway on basal and TGF-β1-mediated *XYLT1* expression (Figure 7). 

A Taqman-based gene expression analysis was performed to examine the role of the TGF-β1-regulated miRNA-21 expression in our myofibroblast cell culture model. We found that the miRNA-21 expression was significantly increased in NHDFs after TGF-β1 supplementation (2.5 ± 0.2-fold, *p* < 0.0001) (Figure 7A). TGF-β1 was shown to be a potent inducer of miRNA-21; therefore, we further examined the direct impact of miRNA-21 on *XYLT1* expression. Since an inverse relationship between the miRNA expression level and its putative target gene was anticipated, we determined the expression level of *SMAD7*, an miRNA-21 target gene confirmed previously [26] and negative regulator of *XYLT1* [7]. Therefore, NHDFs were transfected with an miRNA-21 mimic to resemble the fibrotic miRNA increase. In comparison to the negative control miRNA-transfected cells, miRNA-21 transfection strongly increased the *XYLT1* mRNA expression (1.6 ± 0.3-fold, *p* = 0.003) through a simultaneous decrease of inhibitory *SMAD7* expression (0.5 ± 0.1-fold, *p* = 0.02) (Figure 7B,C). These findings provide a strong argument for the role of the miRNA-21/Smad7 pathway underlying celastrol-mediated effects on *XYLT1* expression.

### 3.6. Amphotericin B Mediates XYLT1 Suppression by Interfering with TGF-β Pathway Components

**Figure 8:**
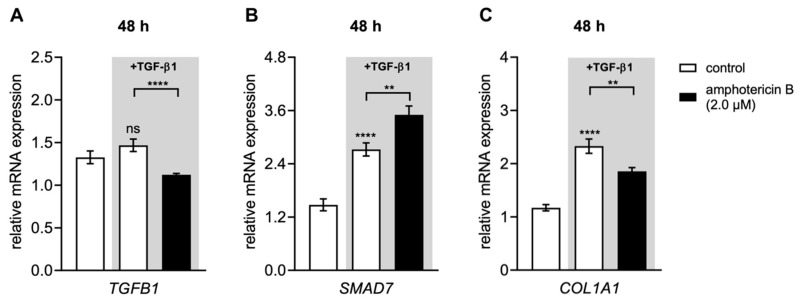
Amphotericin B reduces the TGF-β1-mediated *COL1A1* mRNA expression increase in NHDFs. Human primary dermal fibroblasts (*n* = 3) were cultured the day before the experiment. Cells were treated for 48 h with vehicle only (control), vehicle or 2.0 μM amphotericin B with additional TGF-β1 (5 μg/L) supplementation (highlighted in grey). Relative (**A**) *TGFB1*, (**B**) *SMAD7* and (**C**) *COL1A1* mRNA expression levels were analyzed by qRT-PCR. Data are means ± SEM of three biological and three technical replicates per experiment. Mann–Whitney U test: not significant (ns), *p* < 0.01 (**), *p* < 0.0001 (****).

After showing that amphotericin B is a potent XT-I inhibitor on both a transcriptional and posttranscriptional level, we hypothesized that the anti-fibrotic effect of amphotericin B was mediated by the targeting of downstream TGF-β pathway components, leading to the *XYLT1* mRNA expression decrease observed (Figure 5C–E). We determined the relative *SMAD7*, *TGFB1* and *COL1A1* mRNA expression levels in the NHDFs that were treated with 2.0 μM amphotericin B in the presence or absence of TGF-β1 for 48 h to test this hypothesis (Figure 8).

We could show that the autoinduction of *TGFB1* mRNA expression by TGF-β1 (1.1 ± 0.08-fold, *p* = 0.08) was significantly decreased in the presence of 2 μM amphotericin B (0.8 ± 0.04-fold, *p* < 0.0001). In concordance with these changes, the TGF-β1-induced expression increase of *SMAD7* (1.8 ± 0.2-fold, *p* = 0.002), a known negative regulator of *TGFB1* expression [50], was significantly enhanced in the presence of 2 μM amphotericin B (1.3 ± 0.1-fold, *p* < 0.0001). Regarding the *COL1A1* mRNA expression, we observed a reduction in cells treated with amphotericin B that was not statistically significant compared to controls treated with DMSO (0.7 ± 0.1-fold, *p* = 0.2). In comparison to the cells treated exclusively with DMSO, the presence of TGF-β1 upregulated the *COL1A1* mRNA expression level significantly (0.8 ± 0.06-fold, *p* = 0.0003). This TGF-β1-mediated expression increase could be significantly diminished by the presence of 2.0 μM amphotericin B (0.8 ± 0.06-fold, *p* = 0.008) (Figure 8C).

We assume from these experiments that the amphotericin B-mediated *XYLT1, COL1A1* and *TGFB1* mRNA expression changes under fibrotic conditions were mediated by the simultaneous induction of *SMAD7* expression. 

## 4. Discussion

Myofibroblasts are the key effector cells in excessive ECM synthesis under the pathophysiological conditions that characterize fibrosis [19,51]. TGF-β-driven fibroblast-to-myofibroblast transition was determined by the increased expression and activity of GAG-initiating enzyme XT-I [17,18], corresponding to elevated PG metabolism and increased GAG content in human cells and tissues [52,53]. Thus, modulating downstream TGF-β signaling by the inhibition of XT-I activity could be a promising approach to treat fibrosis. The previous studies performed focused on substrate or end-product analogues such as heparin, GAGs, nucleosides or uridine-derived nucleotides as XT inhibitors. To the best of our knowledge, no studies have been conducted to identify non-substrate-like XT-I inhibitors so far. In the present study, we adapted a previously established enzymatic XT-I activity assay for cell-free screening of an NP-inspired pure compound library with 96 substances regarding their XT-I inhibitory action. We identified four initial compounds with XT-I inhibitory properties: polyene antibiotic amphotericin B, pentacyclic triterpenoid celastrol, tetracycline antibiotic oxytetracycline and natural phenol curcumin. We performed structural- and literature-based analyses and in silico binding analyses to confirm and prioritize the active inhibitory molecules from this primary screening. 

Based on the literature, curcumin is an intensively studied compound with strong assay-interfering properties and, furthermore, is an invalid metabolic panaceas candidate [46,54]. In relation to the enzymatic assay performed here, it can be concluded that curcumin could have interfered by Mg^2+^ or Mn^2+^ ion chelation via the double-activated Michael system or by the binding of the acceptor peptide that has a similar amino acid sequence to silk fibroin [55], leading to false negative screening results. The compound oxytetracycline possesses similar structural features to curcumin, such as several oxygen atoms, which make the chelation of divalent metal ions possible. The C-12 and C-11 oxygen atoms are accepted to be the major binding site for magnesium [56]. The strong ion dependency of the XT-catalyzed reaction was shown previously by Casanova et al. and Müller et al. [30,57]. Taking the molecular docking results and positions into consideration, curcumin and oxytetracycline exhibited the lowest predicted binding affinity to the XT-I protein compared to the compound amphotericin B and celastrol. The binding position for oxytetracycline was predicted to be on the Xylo_C domain (Chimera model #1) or between the Xylo_C and GT_A domain (Chimera model **#**2). Given that mutations in the loop region that mediate the only direct contact between the Xylo_C and GT-A domains did not impair xylose transfer of XT-I [5], we conclude that the discrepancy between the virtual and initial screening data was caused by the reactive structural features. Consequently, the compounds oxytetracycline and curcumin were not further analyzed in the current study. 

The structural- and literature-based analysis of amphotericin B and celastrol showed that amphotericin B is an antibiotic used commonly in tissue culture systems and, therefore, should be suitable for cell-based analysis [58]. Amphotericin B consists of a 38-membered macrolactone ring structure, which is β-glycosylated at the C-19 hydroxyl position with a mycosamine. It could be thought that amphotericin B-protein interactions are based on the formation of hydrogen bonds or by hydrophobic interactions due to the extended conjugated system of the hydrophobic polyene subunits and the high number of hydroxyl groups in the hydrophilic polyol part of the molecule attached [59,60]. Clinical trials report the therapeutic benefit of antioxidant celastrol in inflammatory diseases, whereas cell culture experiments provided the first evidence of the anticancer and neuroprotective properties of celastrol [32,48,49]. Celastrol is structurally a quinone-contained triterpenoid made up of five cyclic rings that can form covalent Michael adducts through the binding of the electrophilic site on quinone methide rings with nucleophilic thiol groups of cysteine residues [61]. Considering the data obtained, the compounds amphotericin B (17) and celastrol (68) were subjected to further analysis. 

We confirmed the results of the initial cell-free screening by the determination of the IC_50_ values, using those to evaluate their inhibitory mode experimentally. Amphotericin B was shown to be an uncompetitive inhibitor regarding the acceptor peptide. This result was consistent with the USCF Chimera [38] AutoDock Vina [37] docking modes predicted. The two docking modes of amphotericin B predicted with the highest binding affinities showed amphotericin B positioned between the Xylo_C and GT-A domains, with the β-glycosylated C-19 mycosamine structure directed towards the GT-A domain. Accordingly, amphotericin B might bind to the enzyme-peptide complex and inhibit consecutive reactions but not the binding of the acceptor peptide to the active site. This hypothesis was underlined by the binding zone analysis, showing that there is a slight overlap between the amphotericin B ring structure around the C-19 position with areas of the UDP-xylose binding site. This uncompetitive inhibitor binding mode towards a substrate is common in enzymes catalyzing bisubstrate reactions [62]. Regarding celastrol, the kinetic studies indicated a competitive inhibitory action of celastrol towards the acceptor peptide. The results were strengthened by the AutoDock Vina [37] predicted docking modes of celastrol. Docking mode **#**1 showed a celastrol binding at the Xylo_C domain, while docking mode **#**2 showed a competitive binding mode on the peptide acceptor side. Given that mutations in the Xylo_C domain did not result in XT-I activity reduction [5], the XT-I activity reduction observed experimentally is likely to have been caused by the competitive binding mode, as illustrated by docking mode **#**2. As has been mentioned above, celastrol is capable of forming covalent adducts with cysteine residues in the protein [61]. Based on the finding that agents (such as *N*-phenyl maleimide) that react with free cysteine residues had no effect on the activity of XT-I [57], we conclude that the celastrol-mediated XT-I activity inhibition observed was possibly not due to covalent Micheal adduct formation. However, a complete analysis of the mechanism of action is required, including an evaluation of other potential inhibition events such as allosteric, partial, tight-binding and time-dependent inhibition, to confirm amphotericin B and celastrol as an uncompetitive and a competitive inhibitor of human XT-I, respectively [62,63]. 

After this initial screen and inhibitor characterization, we performed a cell-based assay to complement the biochemical screening results. The cytotoxic effects of amphotericin B and celastrol on the proliferation of NHDF cells was tested in a proliferation assay. We demonstrated that both compounds were suitable for cell-based analyses at concentrations of 0.5 and 1.0 μM, whereas amphotericin B could be used up to a concentration of 2.0 μM without resulting in proliferation changes. It should be noted that the maximum amphotericin B concentration of 4 μM (3.7 mg/L) tested here already reduced the cellular proliferation significantly and was not used for further analysis. These dose-dependent effects on cellular proliferation are comparable with those reported in the literature [49,58]. Harmsen et al. demonstrated in their in vitro study using osteoblasts and fibroblasts that amphotericin B is lethal to fibroblasts at concentrations of 100 μg/mL, and that this antifungal drug causes sublethal cytotoxicity at 5 and 10 mg/L [58]. The celastrol-mediated inhibition of cellular proliferation in different cell types was tested previously in the concentration range of 0.2 to 10 μM. In agreement with our finding, a time- and concentration-dependent reduction in cellular viability or proliferation was reported [48,49,64]. 

After identifying the optimal amphotericin B and celastrol concentrations for cell-based assays, we characterized their influence on TGF-β1-driven transcription in human cells. We utilized a fibrosis cell culture model established previously and assessed both the direct inhibitor binding to the cellular XT-I protein and the regulation of the *XYLT1* transcription and, thus, influencing the corresponding cellular XT activity. Consistent with the inhibition observed using recombinant CHO cell line-expressed XT-I protein, celastrol was capable of reducing the extracellular XT activity of NHDFs regardless of their *XYLT1* expression. No intracellular XT activity changes were observed, indicating a lack of the membrane permeability of the compound or an unsuited detection time point for intracellular XT activity changes. In agreement with previous results in NHDFs, the intracellular XT activity remained constant over time, while the extracellular XT activity of untreated cells increased over time due to the XT-I protein secretion and accumulation in the cell culture supernatant [7,12]. Consequently, the extracellular XT-I protein secreted can be bound by the inhibitors added exogenously, leading to the XT activity reduction that was observed when compared to samples supplemented with DMSO. The inhibitor compound containing cell culture supernatant was replaced with inhibitor-free media for XT activity detection to overcome this limiting factor of direct inhibitor-protein interaction and observe XT-I activity changes that were originally mediated by transcriptional changes. Using this experimental setup, we found a significant reduction of extracellular XT-I activity quantified 48 h post-amphotericin B treatment and only a slight but not significant reduction of extracellular XT-I activity post treatment with celastrol. The *XYLT1* mRNA expression in 2.0 μM amphotericin B- and 1.0 μM celastrol-treated cells analyzed exhibited a concentration-dependent decrease of TGF-β1-induced *XYLT1* mRNA expression compared to the control. Furthermore, this *XYLT1* transcription regulation seemed to be isoform-specific because the *XYLT2* mRNA expression in the TGF-β1-treated cells was not affected. This correlation of *XYLT1* mRNA expression level and enzyme activity has been shown in numerous studies using TGF-β1-treated human dermal and cardiac fibroblasts [7,18,29,42]. These results clearly show that amphotericin B and, to a smaller extent, celastrol are capable of dual XT-I inhibition via direct protein-ligand binding and *XYLT1* transcription regulation.

There are currently no reports from clinical trials targeting TGF-β signaling in cystic fibrosis (CF) [28], but several studies have already observed a beneficial role of amphotericin B in the treatment of CF or its secondary pulmonary complications. The exact cellular mechanisms underlying those effects have not been fully understood but have been shown as independent of loss-of-function mutation in the cystic fibrosis transmembrane conductance regulator, going beyond the antifungal activity of amphotericin B [65,66]. Since studies of CF patients identified the activation of TGF-β signaling associated with lung fibrosis and myofibroblast differentiation [67], we presumed that amphotericin B could also regulate TGF-β-induced myofibroblast differentiation after sufficient administration [58]. In this context, we have observed a significant reduction of *COL1A1* and *TGFB1* gene expressions through the addition of 2 μM amphotericin B in our fibrosis model with primary NHDFs supplemented with TGF-β1. Furthermore, the TGF-β1-induced expression of inhibitory *SMAD7* further increased in the presence of amphotericin B. Since Smad7 is an important inhibitor of TGF-β superfamily signaling due to its upregulation by TGF-β functioning in a negative feedback loop [50,68], the *XYLT1*, *COL1A1* and *TGFB1* expression decreases observed here might be mediated by this enhanced negative feedback loop. 

It has recently been reported that miRNA-21 is overexpressed during fibrosis and regulates the fibrotic process by modulation of TGF-β1 signaling pathways [24]. To date, nothing is known about the miRNA-21-mediated regulation of XT-I. Therefore, based on the study by Cheng et al. that showed the involvement of the miRNA-21/ERK pathway in celastrol-mediated anti-fibrotic effects in cardiac fibroblasts [48], we analyzed whether miRNA-21 participates in abnormal *XYLT1* regulation under fibrotic conditions. Consistent with the study by Cheng et al. described, we observed an induction of miRNA-21 expression after TGF-β1 stimulation of primary NHDFs in vitro. Performing miRNA-21 overexpression experiments in NHDFs, we identified a novel regulatory mechanism of TGF-β1-driven *XYLT1* expression by the miRNA-21/SMAD7 pathway. These results are in concordance with those of our previous study, demonstrating a suppressive role of miRNA-21 target *SMAD7* [26] in the transcriptional regulation of cytokine-driven *XYLT1* expression [7]. Only a few studies have evaluated the role of miRNAs in TGF-β1-driven *XYLT1* expression regulation so far. Recent studies by our group have revealed an miRNA-145/KLF4- and miRNA-29b/SP1-mediated XT regulatory pathway in human skin fibroblasts [42,69], while Theis et al. demonstrated *XYLT1* to be a direct target of miRNA-133b in the context of murine spinal cord injury [70]. In summary, we were not only able to identify putative XT-I inhibitors in this study but also found first evidence of a regulatory mechanism of TGF-β1-induced *XYLT1* expression by miRNA-21.

## 5. Conclusions

Potent and effective inhibitors of human XT-I could be useful treatment options for cytokine-driven fibrosis. Overall, this investigation identified the two compounds amphotericin B and celastrol as putative non-substrate-based XT-I inhibitors, which were hitherto unknown in this context. Their XT-I activity inhibitory actions seem to be mediated by an uncompetitive or competitive MOI, respectively, regarding the acceptor peptide substrate. In addition, both compounds were capable of decreasing *XYLT1* expression levels and, regarding amphotericin B, subsequent XT activity in myofibroblasts. Due to literature research and cell culture experiments, we suggest a critical role for TGF-β and miRNA-21 pathways in the underlying cellular mechanisms of XT-I inhibition. The results of our study provide a strong rationale for consideration of the putative XT-I inhibitors amphotericin B and celastrol as therapeutic agents in fibroproliferative diseases. 

## Figures and Tables

**Table 1:** Additional primer sequences and annealing temperatures (T_A_) used for the qRT-PCR analysis.

Gene	Primers	T_A_ [°C]	Product Size [bp]
*COL1A1*	5′-GATGTGCCACTCTGACT-3′ 5′-GGGTTCTTGCTGATG-3′	63	151

**Table 2:** Hit compounds of the NP library screening assay that exhibited a relative XT-I activity reduction of more than 50%. The XT-I activities shown are means from one experiment performed in technical duplicates and calculated relative to the XT-I activity of the control.

Compound	Name	XT-I Activity [%]
17	Amphotericin B	25
68	Celastrol	38
74	Oxytetracycline (Terramycin)	17
86	Curcumin	43
0	Dimethyl sulfoxide	100

**Table 3:** Summary of the docking scores and binding interactions of the XT-I protein with compounds 17, 68, 74, and 86, and compound 22 as a control characterized with USCF Chimera 1.14 [38] AutoDock Vina [37]. The two most favored docking modes (Chimera models) for each docked inhibitor compound with the most negative (Score) values are listed and indicated as #1 and #2. The localization of the inhibitor binding site within the XT-I protein domains (inhibitor binding) and the involvement of hydrogen bonding (^H^Bond involvement) for inhibitor-XT-I binding are shown per chimera model predicted.

Compound	Score	Chimera Models	^H^ Bond Involvement	Inhibitor Binding
17	−11.1	Amphotericin B #1	yes	Xylo_C/GT-A domain
17	−10.1	Amphotericin B #2	yes	Xylo_C/GT-A domain
68	−9.7	Celastrol #1	no	Xylo_C domain
68	−9.1	Celastrol #2	no	GT-A domain
74	−8.6	Oxytetracycline #1	yes	Xylo_C domain
74	−8.5	Oxytetracycline #2	yes	Xylo_C/GT-A domain
86	−8.1	Curcumin #1	yes	GT-A domain
86	−7.4	Curcumin #2	yes	Xylo_C domain
22	−6.6	Sulfamethoxazole #1	yes	Xylo_C domain
22	−6.5	Sulfamethoxazole #2	no	Xylo_C domain

## Supplementary Materials

The following are available online at https://www.mdpi.com/2218-273X/10/10/1467/s1: Table S1: Results of the NPs library screening assay. Table S2: Experimental analysis of human XT-I inhibition by celastrol and amphotericin B. Figure S1: Structure of human XT-I complexed with amphotericin B, UDP-d-xylose and the acceptor peptide. Figure S2: *XYLT2* mRNA expression is not affected by inhibitor or cytokine treatment of NHDF. Figure S3: Extracellular XT-I activity reduction by celastrol was not caused by the downregulation of *XYLT1* mRNA expression.
