# Supplementary material for: Identification of Putative Non-Substrate-Based XT-I Inhibitors by Natural Product Library Screening
Source: Biomolecules. 2020 Oct 21;10(10):1467. doi: 10.3390/biom10101467 (PMC7589200; doi:10.3390/biom10101467)
Supplement: Supplementary file 1 [file biomolecules-10-01467-s001.pdf]

biomolecules

MDPI

Supplementary Materials

Table S1. Results of the NPs library screening assay. The inhibitor (50 µM) was incubated with reaction buffer and equally concentrated XT-I protein solutions derived from CHO-K1 pgsA745 cell line complemented with full-length *XYLT1* expressing plasmid. The XT-I activities shown are means from one experiment performed in technical duplicates and calculated relative to the XT-I activity of the negative control sample containing DMSO.

Compound	CAS Number	Molecular Weight	Name	XT-I Activity [%]
**1**	53123-88-9	914.18	Rapamycin (Sirolimus)	70
**2**	56390-09-1	579.98	Epirubicin HCl	95
**3**	18883-66-4	265.22	Streptozotocin (STZ)	101
**4**	364622-82-2	438.52	Doripenem Hydrate	86
**5**	78110-38-0	435.43	Aztreonam	94
**6**	114-07-8	733.93	Erythromycin	105
**7**	2022-85-7	129.09	Flucytosine	93
**8**	72559-06-9	847	Rifabutin	112
**9**	5536-17-4	267.24	Vidarabine	103
**10**	486-66-8	254.24	Daidzein	115
**11**	145-13-1	316.48	Pregnenolone	95
**12**	467214-21-7	653.21	Alvespimycin (17-DMAG) HCl	120
**13**	33419-42-0	588.56	Etoposide	114
**14**	553-21-9	232.32	Costunolide	121
**15**	4759-48-2	300.44	Isotretinoin	86
**16**	4618-18-2	342.3	Lactulose	122
**17**	1397-89-3	924.08	Amphotericin B	25
**18**	317-34-0	420.43	Aminophylline	122
**19**	59-67-6	123.11	Nicotinic Acid	126
**20**	80621-81-4	785.88	Rifaximin	83
**21**	70458-95-6	429.46	Pefloxacin Mesylate	108
**22**	723-46-6	253.28	Sulfamethoxazole	121
**23**	114977-28-5	807.88	Docetaxel	104
**24**	362-07-2	302.41	2-Methoxyestradiol (2-MeOE2)	114
**25**	50-02-2	392.46	Dexamethasone (DHAP)	106
**26**	96036-03-2	383.46	Meropenem	107
**27**	7681-93-8	665.73	Natamycin	95
**28**	54965-21-8	265.33	Albendazole	77
**29**	78613-38-4	353.97	Amorolfine HCl	70
**30**	61379-65-5	877.03	Rifapentine	50
**31**	79902-63-9	418.57	Simvastatin	82
**32**	124832-27-5	360.8	Valaciclovir HCl	74
**33**	127-69-5	267.3	Sulfisoxazole	95
**34**	33069-62-4	853.91	Paclitaxel	79
**35**	2068-78-2	923.04	Vincristine sulfate	87
**36**	86386-73-4	306.27	Fluconazole	75
**37**	501-36-0	228.24	Resveratrol	51
**38**	50-55-5	608.68	Reserpine	89
**39**	128-13-2	392.57	Ursodiol	98
**40**	56-75-7	323.13	Chloramphenicol	87
**41**	13292-46-1	822.94	Rifampin	77
**42**	59277-89-3	225.2	Aciclovir	71

**43**	110871-86-8	392.4	Sparfloxacin	89
**44**	62997-67-5	1056.24	Nystatin (Fungicidin)	52
**45**	187235-37-6	359.26	PA-824	75
**46**	56180-94-0	645.6	Acarbose	92
**47**	112811-59-3	375.39	Gatifloxacin	98
**48**	220620-09-7	585.65	Tigecycline	59
**49**	91832-40-5	395.41	Cefdinir	72
**50**	59-87-0	198.14	Nitrofural	104
**51**	63-74-1	172.2	Sulfanilamide	118
**52**	117467-28-4	620.72	Cefditoren Pivoxil	104
**53**	83905-01-5	748.98	Azithromycin	109
**54**	98-92-0	122.12	Nicotinamide (Vitamin B3)	116
**55**	100986-85-4	361.37	Levofloxacin	111
**56**	73-31-4	232.28	Melatonin	99
**57**	63968-64-9	282.33	Artemisinin	93
**58**	446-72-0	270.24	Genistein	94
**59**	165800-03-3	337.35	Linezolid	103
**60**	23593-75-1	344.84	Clotrimazole	101
**61**	58-61-7	267.24	Adenosine	103
**62**	50-23-7	362.46	Hydrocortisone	122
**63**	68-35-9	250.28	Sulfadiazine	108
**64**	773-76-2	214.05	Chloroxine	121
**65**	68-19-9	1355.37	Vitamin B12	94
**66**	58-27-5	172.18	Menadione	106
**67**	25316-40-9	579.98	Doxorubicin (Adriamycin) HCl	113
**68**	34157-83-0	450.61	Celastrol	38
**69**	15291-77-7	424.4	Ginkgolide B	100
**70**	137234-62-9	349.31	Voriconazole	94
**71**	62893-19-0	645.67	Cefoperazone	83
**72**	302-79-4	300.4	Tretinoin	84
**73**	57-83-0	314.46	Progesterone	113
**74**	79-57-2	460.43	Oxytetracycline (Terramycin)	17
**75**	474-25-9	392.57	Chenodeoxycholic Acid	113
**76**	443-48-1	171.15	Metronidazole	109
**77**	298-81-7	216.19	Methoxsalen	107
**78**	51-21-8	130.08	Fluorouracil (5-Fluoracil, 5-FU)	137
**79**	152044-53-6	493.66	Epothilone A	119
**80**	103060-53-3	1620.67	Daptomycin	86
**81**	70356-03-5	385.82	Cefaclor	71
**82**	651-06-9	280.3	Sulfameter	126
**83**	53-16-7	270.37	Estrone	120
**84**	50-28-2	272.38	Estradiol	128
**85**	536-33-4	166.24	Ethionamide	130
**86**	458-37-7	368.38	Curcumin	43
**87**	65899-73-2	387.71	Tioconazole	136
**88**	22832-87-7	479.14	Miconazole Nitrate	149
**89**	144-82-1	270.33	Sulfamethizole	111
**90**	68373-14-8	233.24	Sulbactam	138
**91**	122-11-2	310.33	Sulphadimethoxine	132
**92**	10212-25-6	261.66	Cyclocytidine HCl	142
**93**	2922-28-3	171.59	Adenine HCl	146
**94**	404-86-4	305.41	Capsaicin (Vanilloid)	135

**95**	15291-75-5	408.4	Ginkgolide A	150
**96**	9041-93-4	1512.62	Bleomycin sulfate	149
**0**	67-68-5	78.13	Dimethyl sulfoxide	100

Table S2. Experimental analysis of human XT-I inhibition by celastrol and amphotericin B. K_m and V_{max} values shown were calculated from nonlinear regression (Michaelis-Menten plot) and the corresponding Y-intercept and slope values from the simple linear regression (Lineweaver-Burk plot). Data are means ± SEM of triplicate data points per experiment.

**Compound.**	**Michaelis-Menten plot**		**Lineweaver-Burk plot**	
DMSO	V_{max} [AU]	12124 ± 626	Y-intercept	$7.8 \pm 1.3 \cdot 10^{-5}$
	K_m [µM]	31.2 ± 3	slope	$2.6 \cdot 10^{-3} \pm 5.09 \cdot 10^{-5}$
Celastrol	V_{max} [AU]	11826 ± 551	Y-intercept	$7.1 \pm 1.1 \cdot 10^{-5}$
	K_m [µM]	53.3 ± 3	slope	$4.5 \cdot 10^{-3} \pm 7.4 \cdot 10^{-5}$
Amphotericin B	V_{max} [AU]	10031 ± 388	Y-intercept	$9.1 \pm 1.5 \cdot 10^{-5}$
	K_m [µM]	27.5 ± 3	slope	$2.8 \cdot 10^{-3} \pm 9.6 \cdot 10^{-5}$

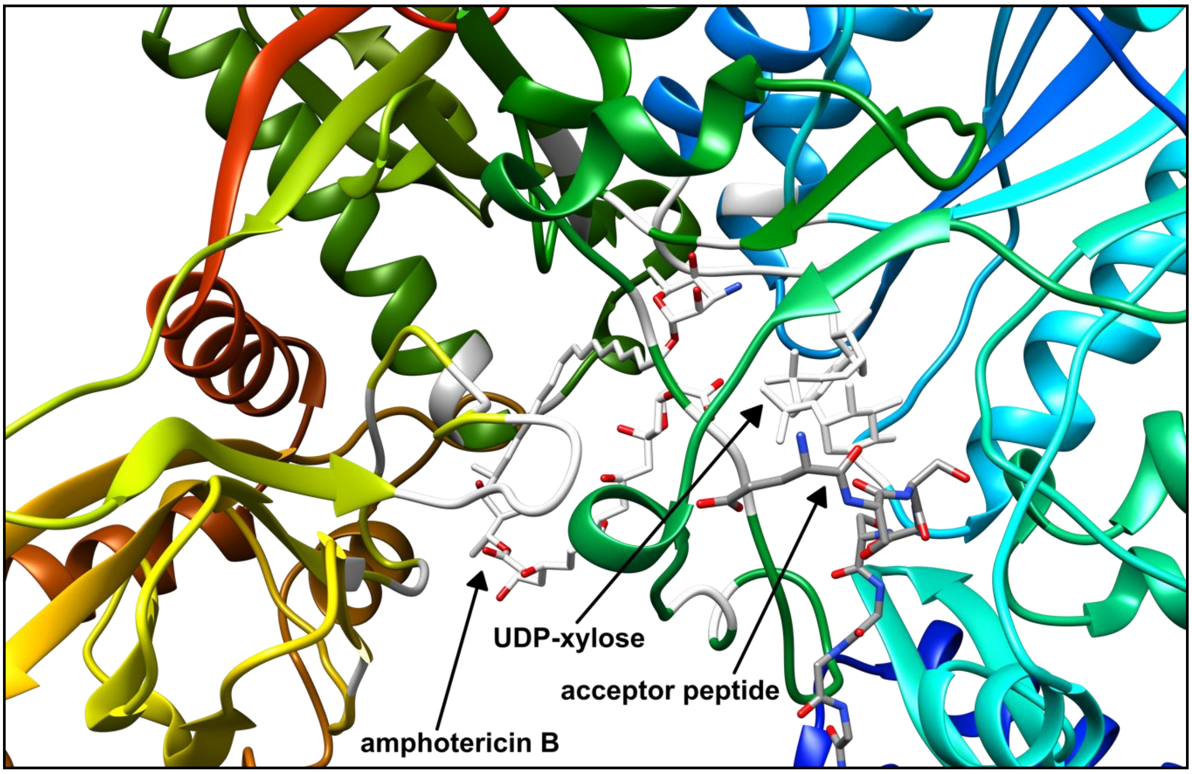

Figure S1. Structure of human XT-I complexed with amphotericin B, UDP-D-xylose and the acceptor peptide. Crystal structure of human XT-I [5] (rainbow colored from the N terminus (blue) to C terminus (red)) complexed with the chimera models #**2** of amphotericin B (white colored), UDP-D-xylose and modified acceptor peptide (atoms: C (grey), N (blue), O (red), P (orange)) are shown in stick representation. All atoms and bonds that meet the criteria < 5.0 Å from amphotericin B were colored in white.

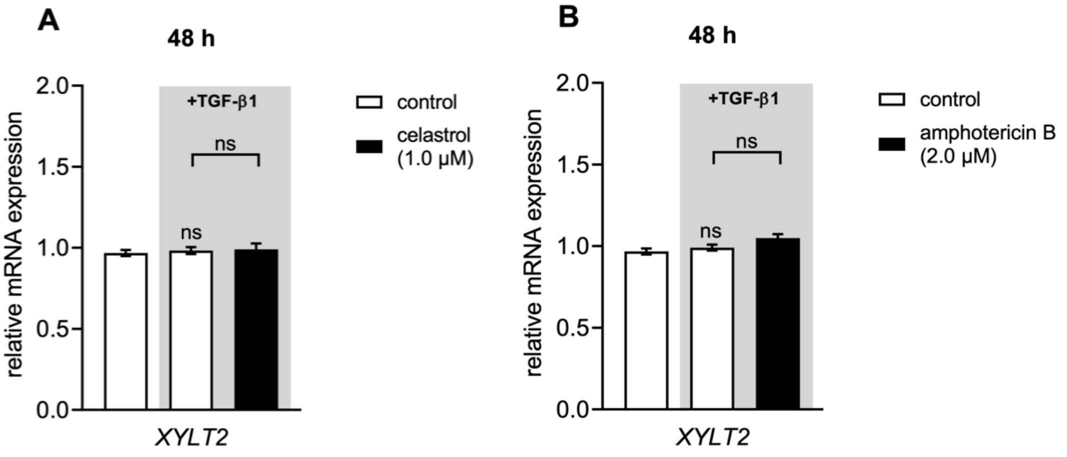

Figure S2. *XYLT2* mRNA expression is not affected by inhibitor or cytokine treatment of NHDF. Human primary dermal fibroblasts (n = 3) were cultured the day before the experiment. Cells were treated for 48 h with vehicle only (control), vehicle or (**A**) 1.0 µM celastrol or (**B**) 2.0 µM amphotericin B with additional TGF-β1 (5 µg/L) supplementation (highlighted in grey). Relative *XYLT2* mRNA expression levels were analyzed by quantitative real-time PCR. Data are means ± SEM of three biological and three technical replicates per experiment. Mann-Whitney U test: not significant (ns).

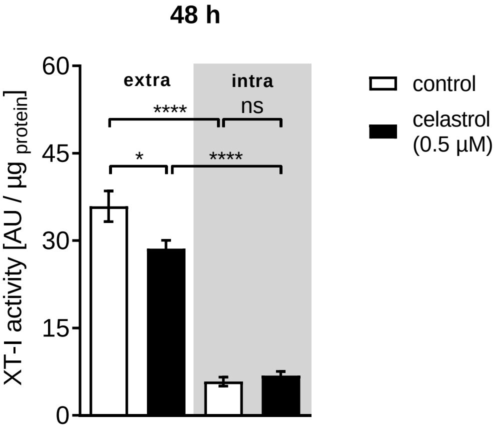

Figure S3. Extracellular XT-I activity reduction by celastrol was not caused by the downregulation of *XYLT1* mRNA expression. Human primary dermal fibroblasts (n = 3) were cultured the day before the experiment. Cells were treated with vehicle (control) or 0.5 µM celastrol for 48 h. Intracellular XT activity (intra, grey) was determined from the cell lysates and the corresponding supernatants were utilized for extracellular XT activity (extra) determination by UPLC-ESI-MS/MS XT-I assay. The XT activity is expressed as arbitrary units (AU) per µg of protein in 1 mL sample. Data are means ± SEM of three biological and three technical replicates per experiment. Mann-Whitney U test: not significant (ns), $p < 0.05$ (*), $p < 0.0001$ (****).
